# Aroid scarabs in the genus *Peltonotus* Burmeister (Coleoptera, Scarabaeidae, Dynastinae): key to species and new distributional data

**DOI:** 10.3897/zookeys.320.5352

**Published:** 2013-07-31

**Authors:** Mary Liz Jameson, Alain Drumont

**Affiliations:** 1 Department of Biological Sciences, Wichita State University, Wichita, Kansas, 67260–0026, USA; 2 Institut Royal des Sciences Naturelle de Belgique, B-1000 Brussels, Belgium

**Keywords:** Edible insects, Palearctic region, Sino-Japanese region, Araceae, dynamic mapping

## Abstract

The southeast Asian scarab beetle genus *Peltonotus* Burmeister (Scarabaeidae, Dynastinae, Cyclocephalini) is reviewed. New country records for *Peltonotus morio* Burmeister (Myanmar and Vietnam), *Peltonotus nasutus* Arrow (southern China and Cambodia), and *Peltonotus favonius* Jameson and Wada (Myanmar) are reported, including a new record in the Palearctic/Sino-Japanese biogeographic region. The first female specimen of *Peltonotus favonius* is described. Biological associations with aroid inflorescences are reviewed, and human consumption of *Peltonotus* beetles is reported. A key to all species, paralectotype designations for *Peltonotus nasutus*, diagnoses, and distributions using dynamic mapping tools are included.

## Introduction

The scarab beetle genus *Peltonotus* Burmeister (Scarabaeidae, Dynastinae) includes 25 species that are distributed in forest habitats in Southeast Asia and that are associated with aroid inflorescences (Araceae) (Jameson and Wada 2004). Adult beetles use inflorescences as sites for mating and feeding, and they serve as pollinators (Moore and Jameson in press, Maia et al. 2012). Species in the genus are intimately tied to host aroids and their forest habitats, and we predict that many species await discovery. Members of the genus form a natural group based on a unique, articulated maxillary tooth. The first monograph for the genus (Jameson and Wada 2004) included 19 species; since this time, six additional species have been described (Jameson and Wada 2009, Jameson and Jakl 2010), a 30% increase in species diversity.

Identification of species in the genus *Peltonotus* is hampered by sexual dimorphism that makes association of conspecific sexes difficult, absence of male or female specimens for some species, rarity of some species (perhaps due to brief activity patterns and host plant phenology), and color variability within species. For this reason, we amalgamate existing keys into one identification guide for males and females and provide diagnoses.

Species of *Peltonotus* are associated with aroid inflorescences (Araceae) (Jameson and Wada 2004). However, in comparison to the abundant research on aroid and scarab beetle interactions in the New World tropics (e.g., Gibernau et al. 2010; Maia et al. 2012; Young 1988), little research is being conducted on *Peltonotus* and aroids in the Old World. *Peltonotus malayensis* Arrow is associated with inflorescences of the climbing aroid, *Epipremnum falcifolium* Engl. (Araceae) (Jameson and Wada 2004). Male and female beetles (as well as many small beetles and arthropods) have been reported around the base of the spathe where adult *Peltonotus malayensis* were observed mating and feeding. Inflorescences of the cultivated aroid, *Amorphophallus paeoniifolius* (Dennst.) Nicolson (Araceae), attract aggregations of *Peltonotus nasutus* Arrow (Grimm 2009). This plant (also called the elephant foot yam or corpse plant) grows on the forest floor in dappled shade or in the open sun in secondary forest or highly disturbed areas. The large flower (up to 40 cm) smells like a rotting dead animal and deceptively attracts insects that may serve as pollinators (Schiestl and Dötterl 2012) including the carrion scarabs *Phaeochrous dissimilis* Arrow, *Phaeochrous emarginatus* Laporte, and *Phaeochrous intermedius* Pic (all Scarabaeoidea, Hybosoridae), and the aroid scarab *Peltonotus nasutus* (Grimm 2009). Additional research on aroids and *Peltonotus* species is needed in order to clarify plant-insect interactions including evolution, ecology, and pollination.

In addition to being associated with aroid inflorescences, adults are attracted to lights at night, and some have been collected in malaise traps. Adults may have short seasonal activity patterns. Some adults have been recorded for only two nights during season-long, intensive collecting efforts. Larvae are not known for any species in the genus.

Survey efforts and collecting in Southeast Asia have provided new distributional data for species in the genus, thus yielding a clearer understanding of distribution patterns. Herein, we report new distributional data for three species of *Peltonotus*. Because identification of species requires use of three publications (Jameson and Wada 2004, Jameson and Wada 2009, Jameson and Jakl 2010), we provide a comprehensive key to all species in the genus, short diagnoses, new paralectotype designations for *Peltonotus nasutus*, and maps with associated files for dynamic mapping capabilities.

## Material and methods

Characters and specimens were examined using a dissecting microscope (6–48× magnification) and fiber-optic illumination. Digital images of specimens and structures were captured using the Leica Application Suite V3.8. Images were edited in Adobe Photoshop CS2 (background removed, contrast manipulated). In the absence of images for some specimens, illustrations are used. Specimen localities that were not recorded in latitude and longitude on original labels were translated using GoogleEarth (www.google.com/earth/index.html) or by using the Global Gazetteer Version 2.2 (www.fallingrain.com/world/). It should be noted that older localities have a wide margin of error, and their lack of precision is not conducive to ecological or niche modeling. Maps were generated by entering these data into Microsoft Excel 2008 and uploaded to EarthPoint (www.earthpoint.us/Excel-ToKml.aspx) and GoogleEarth (Appendix 1). These mapping tools allow for interactive mapping and addition of data by subsequent users. Locality information in species treatments is recorded with the country in bold letters, followed by the state/province/district, and the specific locality in parentheses.

This work unifies some character state definitions (e.g., form of labrum, male protibial teeth, female elytral epipleural pillow) previously used for identification of *Peltonotus* species. Species are characterized by combinations of characters including the form of the labrum (weakly sinuate, bi-emarginate/broadly emarginated, or deeply bilobed) (Figs 20–24), mentum apex and second labial palpomere (compared with palpomere 1) (Figs 25–35), mala of maxilla with or without thickened and strongly flattened setae (“lamellate setal brush”) (Figs 36–44), stipes of maxilla with or without curly setae (Figs 36–44), male protibia tri- or bidentate (Figs 45–49), form of male protarsomeres (Figs 50–54), form of the male parameres (Figs 55–72), and form of the female epipleuron in ventral view in relation to the position of the metacoxa (Figs 73–91). Expansions of the female elytral epipleuron may have an inflated area (or pillow) in dorsal view (Moore 2012). Setae are important for species diagnosis and are defined as minute if they are less than 0.2 mm, short if between 0.2–0.5 mm, moderately long if between 0.5–1.0 mm, and long if between 1.0–2.0 mm (as measured with an ocular micrometer). Punctures may lack setae, possess one seta (unisetigerous), or possess multiple setae (multisetigerous). Male parameres are highly asymmetrical, and we elected to illustrate the lateral view that best assists in identification.

We follow the phylogenetic species concept (Wheeler and Platnick 2000) that states that “A species is the smallest aggregation of (sexual) populations or (asexual) lineages diagnosable by a unique combination of character states.” Specimens examined for this research are deposited in the following institutions and private collections: the Institut Royal des Sciences Naturelle de BelgiqueIRSNB, the Alain Drumont Collection, Brussels, Belgium; the Masayuki Fujioka Collection, Tokyo, JapanFUJI; the Museum National d’Histoire Naturelle, Paris, FranceMNHN; Andreas Reichenbach Collection, Leipzig, GermanyAREC; the Mary Liz Jameson collection, Wichita, KansasMLJC; the Shinji Nagia Collection (Nagano, Japan); and The Natural History Museum, London, EnglandBMNH.

### New distributional records, human consumption, and paralectotype designations for *Peltonotus nasutus*

*Peltonotus nasutus* (Figs 14–15) is the most distinctive species within the genus *Peltonotus* due to its large body size (~20 mm), tubercle at the apex of the clypeus in the male (Fig. 23), and greatly enlarged protibial claw in the male (Fig. 52).

Large aggregations of adults (over 100) have been found in association with the large, fetid-smelling aroids in the genus *Amorphophallus* (Grimm 2009; label data at BMNH). In Thailand, the stench of flowering *Amorphophallus paeoniifolius* attracts a profusion of *Peltonotus nasutus* individuals that serve to pollinate the inflorescence. Seventy eight specimens were recorded in one flower, and these were collected, fried with fish sauce and salt, and then consumed by the Karen-speaking tribe in the Tak province in northern Thailand (Danell 2010). Thai people consume more insects per capita than other people and cultures (Chen et al. 1998), and this beetle species is a new record for human consumption.

**Figures 1–12.**
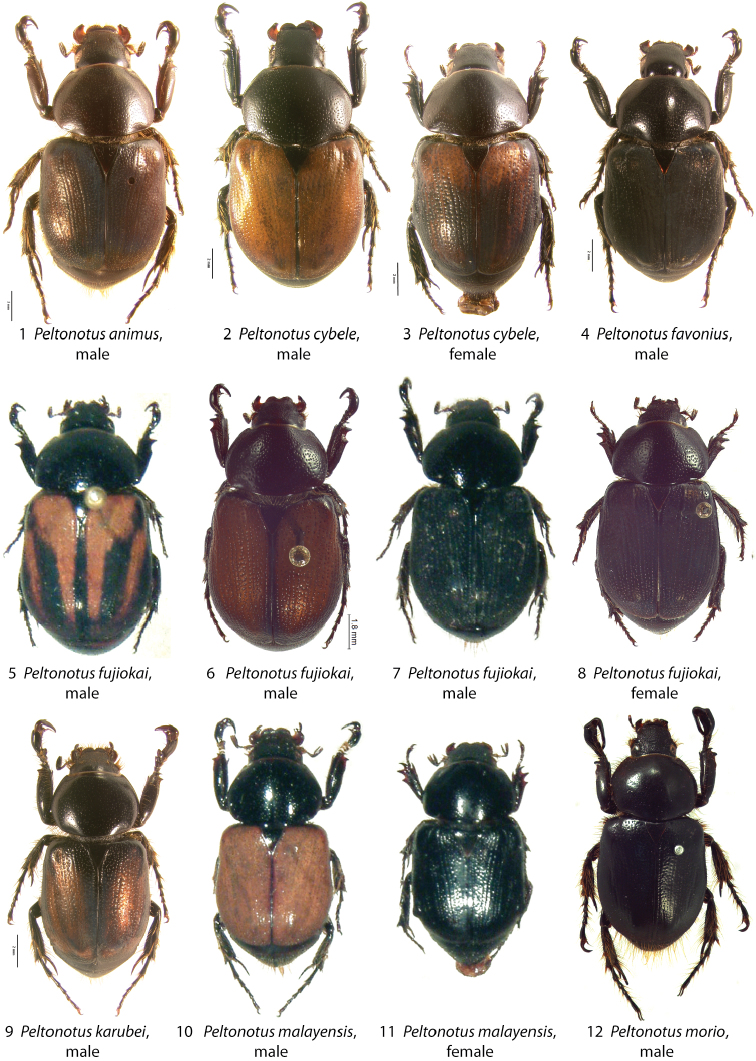
*Peltonotus* species dorsal habitus. **1** *Peltonotus animus*, male **2–3** *Peltonotus cybele*, male and female (respectively) **4** *Peltonotus favonius*, male **5–7** *Peltonotus fujiokai*, males (showing variation) **8** *Peltonotus fujiokai*, female **9** *Peltonotus karubei*, male **10–11** *Peltonotus malayensis*, male and female (respectively) **12** *Peltonotus morio*, male.

**Figures 13–19.**
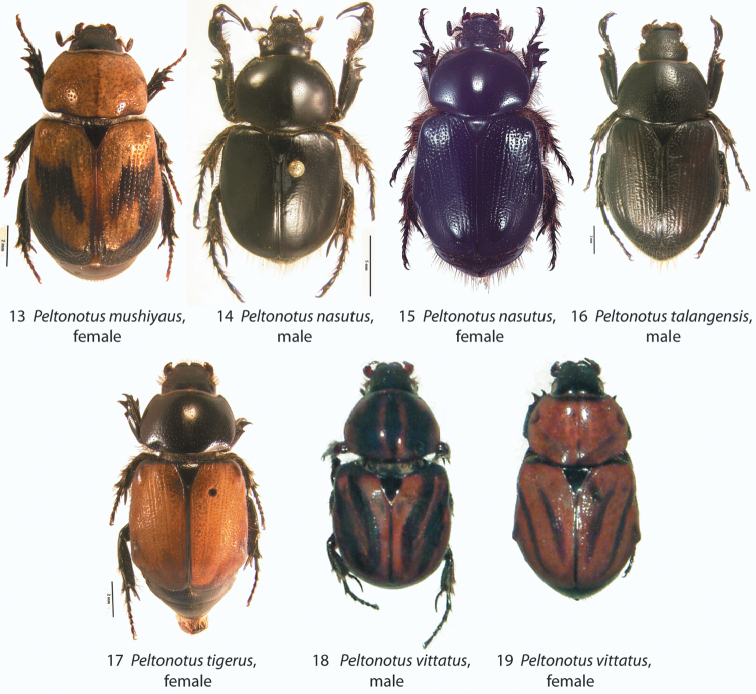
*Peltonotus* species dorsal habitus. **13** *Peltonotus mushiyaus*, female **14–15** *Peltonotus nasutus*, male and female (respectively) **16** *Peltonotus talangensis*, male **17** *Peltonotus tigerus*, female **18–19** *Peltonotus vittatus*, male and female (respectively).

**Figures 20–24.**
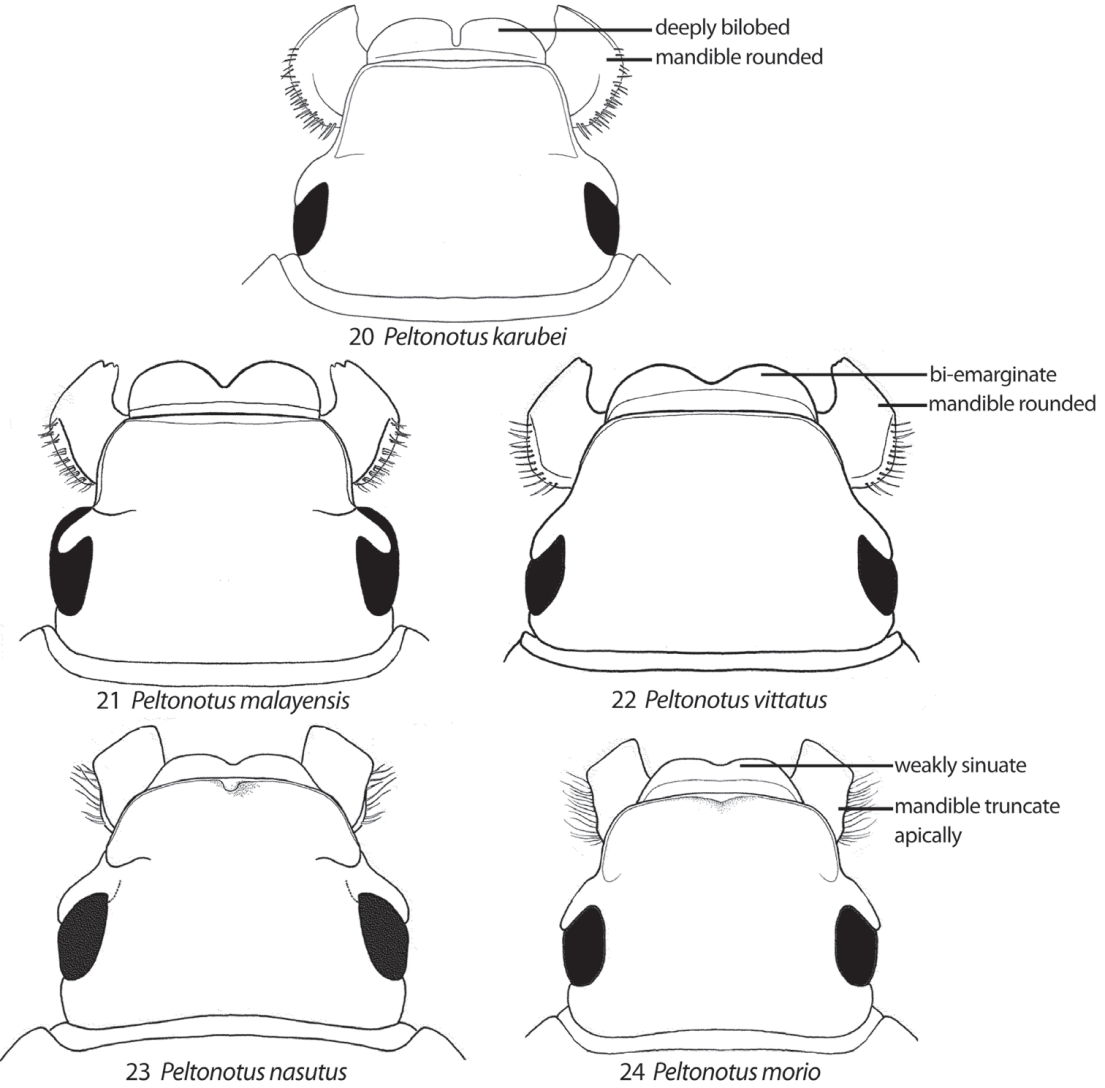
Head (dorsal view) showing characters of the labrum, mandible, and clypeus. **20** *Peltonotus karubei* (apex of labrum deeply bi-lobed; apex of mandible rounded laterally) **21** *Peltonotus malayensis* (apex of labrum bi-emarginate; apex of mandible rounded laterally) **22** *Peltonotus vittatus* (apex of labrum bi-emarginate; apex of mandible rounded laterally) **23** *Peltonotus nasutus* (apex of labrum weakly sinuate; apex of mandible quadrate laterally with broadly truncate apex; apex of clypeus with weak tubercle in male) **24** *Peltonotus morio* (apex of labrum weakly sinuate; apex of mandible quadrate laterally with broadly truncate apex; apex of clypeus without tubercle in male).

**Figures 25–35.**
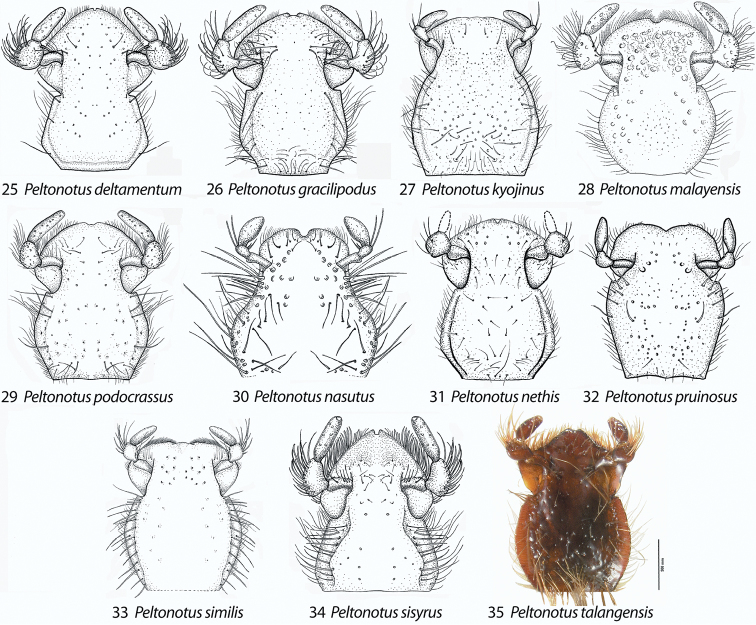
Mentum, ventral view, showing form of apical half of mentum and form of labial palpomere 2 (in comparison to palpomere 1). **25** *Peltonotus deltamentum* **26** *Peltonotus gracilipodus* **27** *Peltonotus kyojinus* **28** *Peltonotus malayensis* **29** *Peltonotus podocrassus* **30** *Peltonotus nasutus* **31** *Peltonotus nethis* **32** *Peltonotus pruinosus* **33** *Peltonotus similis* **34** *Peltonotus sisyrus* **35** *Peltonotus talangensis*.

**Figures 36–44.**
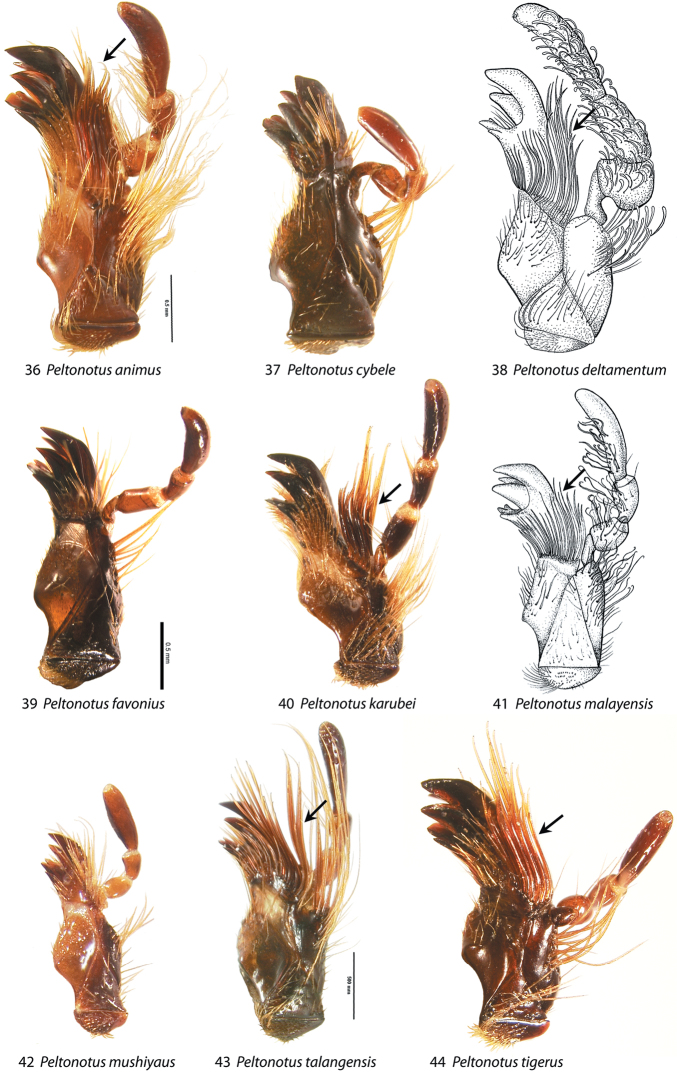
Maxilla, ventral view, showing mala with or without lamellate setal brush (setae thick and strongly flattened), and showing stipes with or without setae curly at apices. **36** *Peltonotus animus* **37** *Peltonotus cybele* **38** *Peltonotus deltamentum* **39** *Peltonotus favonius* **40** *Peltonotus karubei* **41** *Peltonotus malayensis* **42** *Peltonotus mushiyaus* **43** *Peltonotus talangensis* **44** *Peltonotus tigerus*. Arrows indicate lamellate setal brush.

**Figures 45–54.**
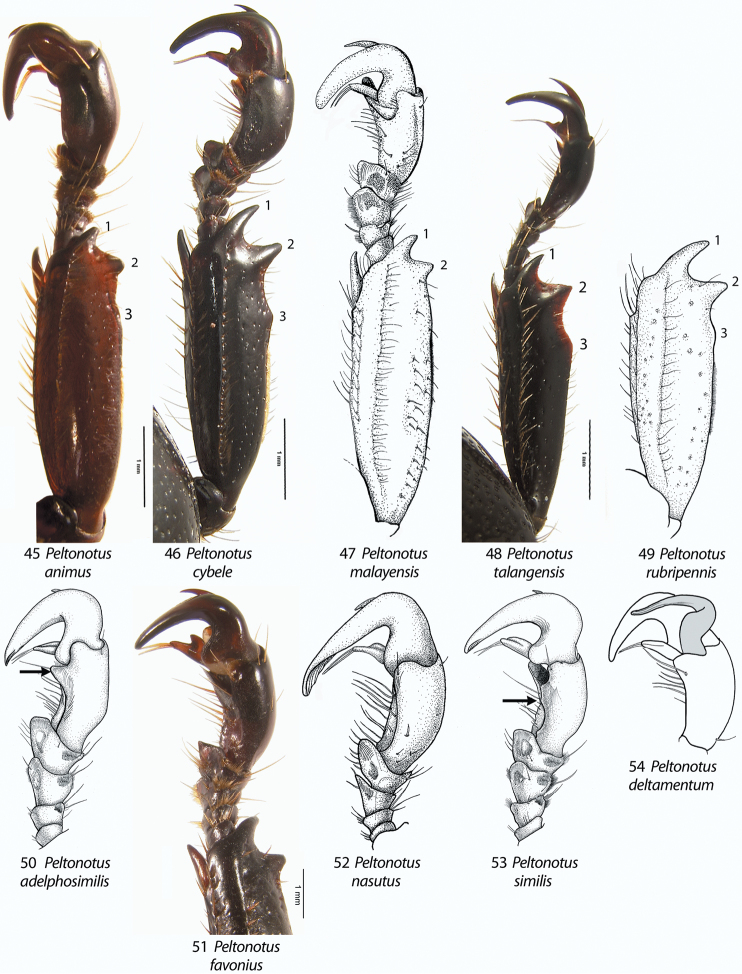
Male prolegs, dorsal view (**45–49**), male protarsomeres, dorsal view (**50–53**), and male protarsomere 5, ventral view (**54**), of *Peltonotus*. **45** *Peltonotus animus* (male protibia tridentate with basal tooth obsolete) **46** *Peltonotus cybele* (male protibia tridentate with basal tooth well developed) **47** *Peltonotus malayensis* (male protibia bidentate) **48** *Peltonotus talangensis* (male protibia tridentate with basal tooth well developed) **49** *Peltonotus rubripennis* (male protibia tridentate with basal tooth weakly developed) **50** *Peltonotus adelphosimilis* (arrow showing protarsomere 5 of male with internoapical protuberance) **51** *Peltonotus favonius* (male protibia bidentate) **52** *Peltonotus nasutus* (male protibial claw greatly enlarged) **53** *Peltonotus similis* (arrow showing protarsomere 5 of male with internomedial protuberance) **54** *Peltonotus deltamentum* (male proclaw strongly arcuate in ventral view).

**Figures 55–60.**
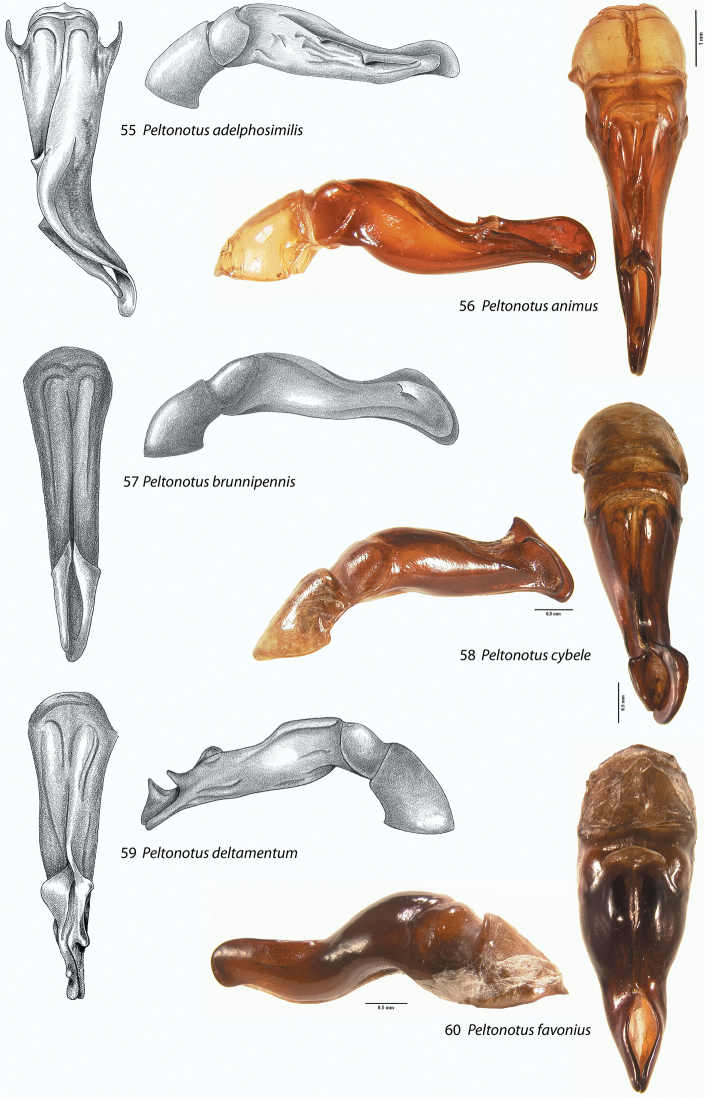
Male parameres (with or without phallobase), dorsal and lateral views, in *Peltonotus*. Male parameres are highly asymmetrical, and we illustrate the lateral view that best assists in identification. **55** *Peltonotus adelphosimilis* **56** *Peltonotus animus* **57** *Peltonotus brunnipennis* **58** *Peltonotus cybele* **59** *Peltonotus deltamentum* **60** *Peltonotus favonius*.

**Figures 61–66.**
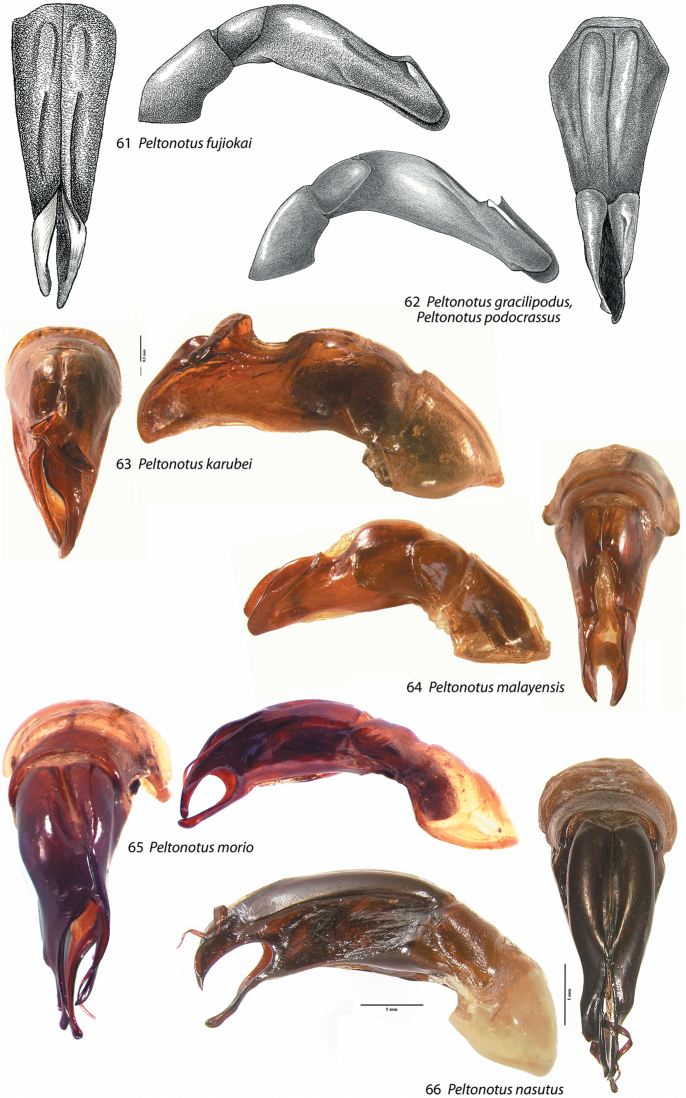
Male parameres (with or without phallobase), dorsal and lateral views, in *Peltonotus*. Male parameres are highly asymmetrical, and we illustrate the lateral view that best assists in identification. **61** *Peltonotus fujiokai* **62** *Peltonotus gracilipodus* and *Peltonotus podocrassus* **63** *Peltonotus karubei* **64** *Peltonotus malayensis* **65** *Peltonotus morio* **66** *Peltonotus nasutus*.

**Figures 67–72.**
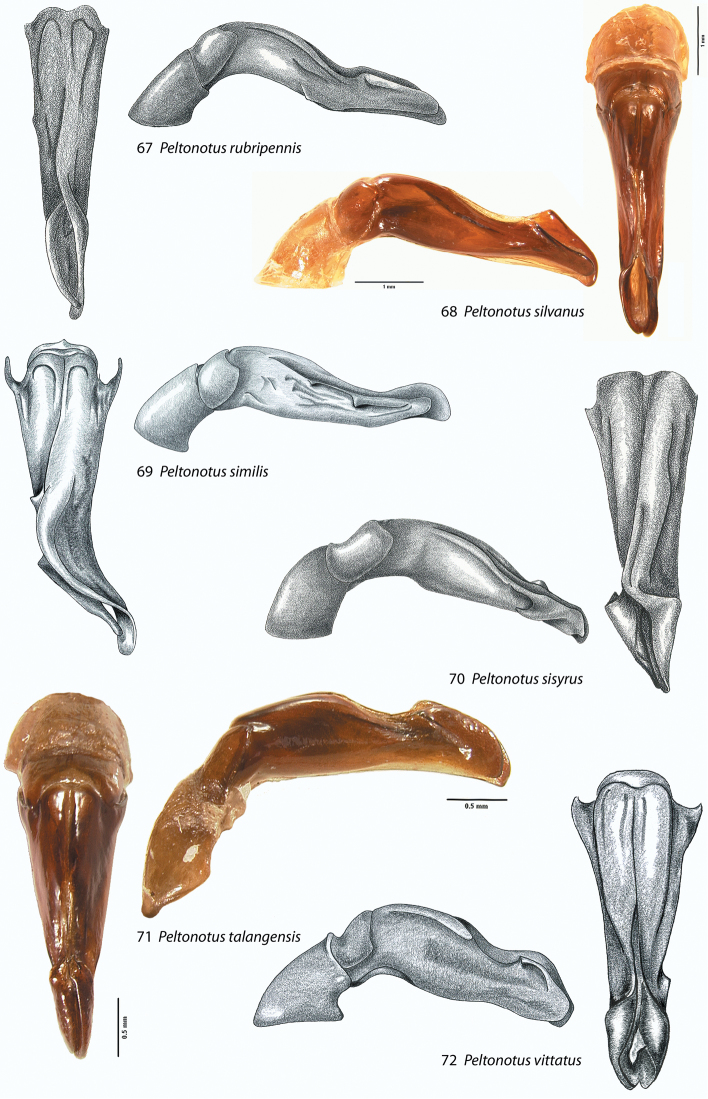
Male parameres (with or without phallobase), dorsal and lateral views, in *Peltonotus*. Male parameres are highly asymmetrical, and we illustrate the lateral view that best assists in identification. **67** *Peltonotus rubripennis* **68** *Peltonotus silvanus* **69** *Peltonotus similis* **70** *Peltonotus sisyrus* **71** *Peltonotus talangensis* **72** *Peltonotus vittatus*.

**Figures 73–87.**
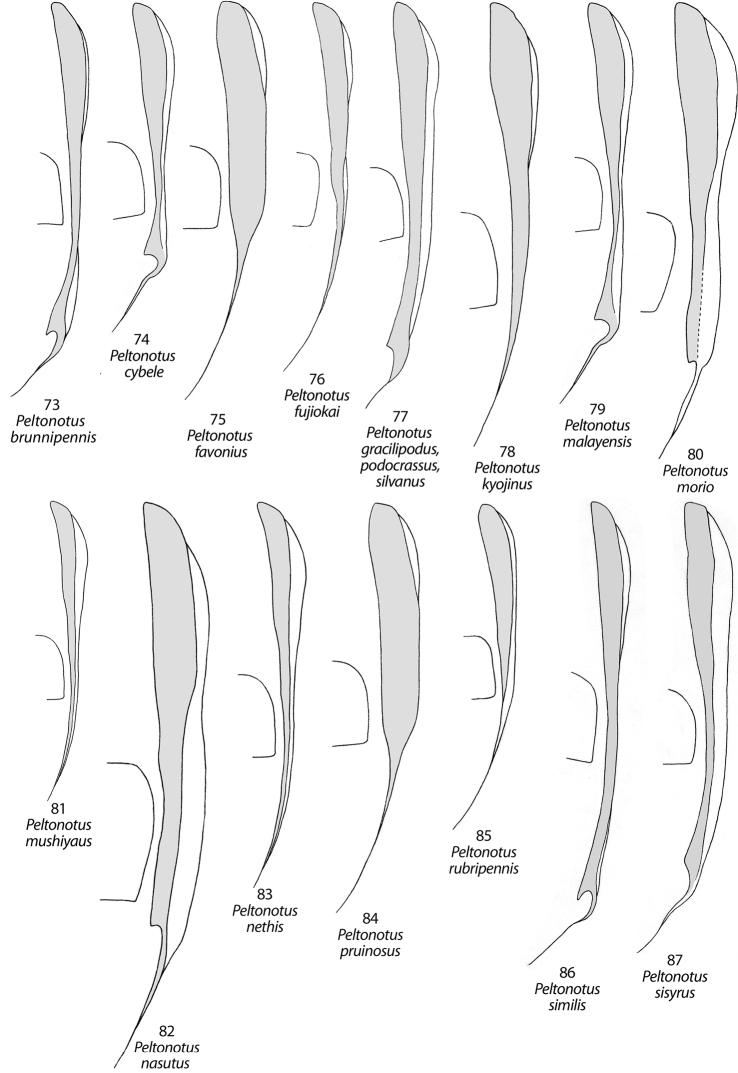
Female elytral epipleuron (gray, ventral view) and position relative to metacoxa in *Peltonotus*. **73** *Peltonotus brunnipennis* **74** *Peltonotus cybele* **75** *Peltonotus favonius* **76** *Peltonotus fujiokai* **77** *Peltonotus gracilipodus*, *Peltonotus podocrassus* and *Peltonotus silvanus* **78** *Peltonotus kyojinus* **79** *Peltonotus malayensis* **80** *Peltonotus morio* **81** *Peltonotus mushiyaus* **82** *Peltonotus nasutus* **83** *Peltonotus nethis* **84** *Peltonotus pruinosus* **85** *Peltonotus rubripennis* **86** *Peltonotus similis* **87** *Peltonotus sisyrus*

**Figures 88–91.**
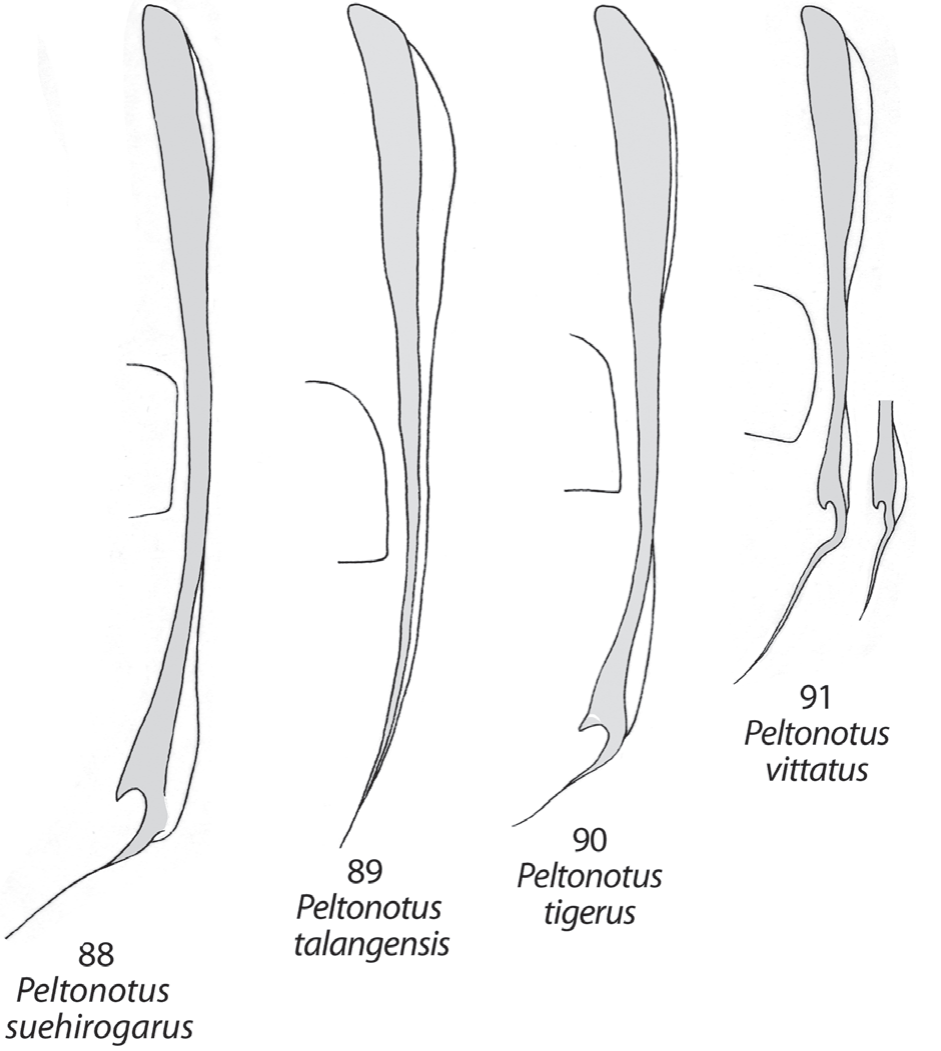
Female elytral epipleuron (gray, ventral view) and position relative to metacoxa in *Peltonotus*. **88** *Peltonotus suehirogarus* **89** *Peltonotus talangensis* **90** *Peltonotus tigerus* **91** *Peltonotus vittatus*.

**Figure 92.**
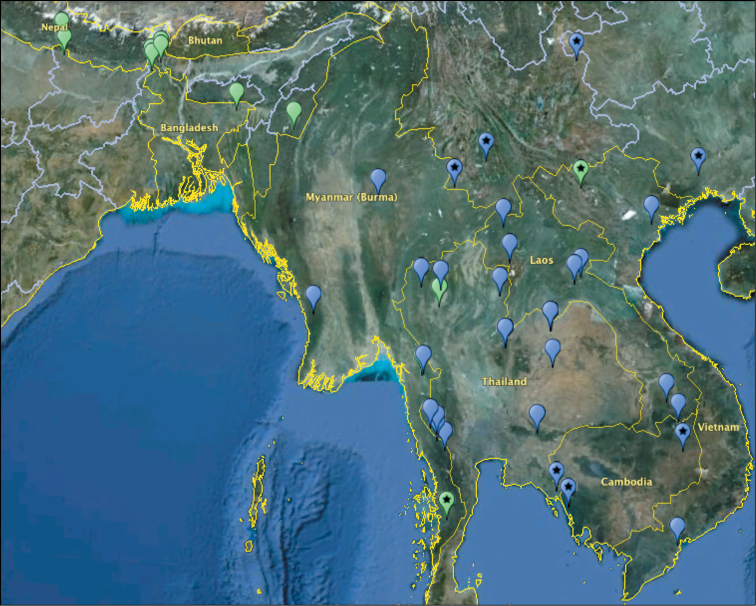
Distribution of *Peltonotus morio* (green icon) and *Peltonotus nasutus* (blue icon) in southeast Asia. Icons with stars indicate new country records for each species. Map was generated using data in **Table 1**.

The species is distributed in Myanmar, Thailand, Laos, and Vietnam (Jameson and Wada 2004; Li et al. 2012) (Fig. 92). Adults inhabit deciduous dipterocarp forests between 100–800 m elevation and have been collected at mercury vapor light traps. Examination of additional specimens provided **new country records** for *Peltonotus nasutus* in Cambodia and China. This species was not previously recorded as occurring in the Palaearctic region (as defined by Löbl and Smetana 2003). These records demonstrate that the species occurs in the Guangxi and Guizhou provinces of southern portion of China in what is considered the Palaearctic biogeographic region (Löbl and Smetana 2003) or the Sino-Japanese biogeographic region (Holt et al. 2013). **New Country Record: CHINA** (6 males, 2 females deposited in Drumont Collection; AREC): Guangxi Zhuang Autonomous Region (Guangxi), Guizhou (Weining, Mt. Ping-Qing-Liang-Zi), Yunnan (Jinggu, Mt. Longtanshan; Menglian, Mt. Daheishan). Specimens were collected from May to July: May (1), June (3), July (4). **New Country Record: CAMBODIA** (9 males, 12 females deposited at IRSNB): Pursat (Phnom Samkos Wildlife Sanctuary), Ratanakiri (Phumi Kalai Thum), Pailin (Pailin). Specimens were collected from April to June and November: April (3), May (2), June (3), November (13). The new country record in Weining, China extends the known range of the species over 600 km north.

**Table 1.** *Peltonotus* Locality Table. Locality information for *Peltonotus morio* and *Peltonotus nasutus*. The Appendix file can be used for dynamic mapping using EarthPoint and GoogleEarth.

**Latitude, Longitude**	**Species name**	**Collection or Reference**	**Locality Information**
16°40'27"N, 98°17'59"E	*Peltonotus morio*	FUJI	S. Burma, Mt. Dawna, V.1992, 1 male, ele. 763m, NEW COUNTRY RECORD
12°05'N, 99°00'E	*Peltonotus morio*	FUJI	S. Burma, Tenasserim, V.1992, 1 female, NEW COUNTRY RECORD
26°52'41"N, 88°17'25"E	*Peltonotus morio*	BMNH	India, Kurseong Div., Lat Panchar, 4000 ft., VI.1934, 6 specimens, Col. Champion
27°39'N, 84°19'E	*Peltonotus morio*	Dhoj et al. 2009	Nepal, Chitwan Central region, Gunjanagar, 230 m
27°39'N, 84°21'E	*Peltonotus morio*	Dhoj et al. 2009	Nepal, Rampur, 230 m, amid maize-maize-vegetables in sandy soil from farming sites.
22°29'N, 103°57'E	*Peltonotus morio*	IRSBN	Vietnam, Lao Cai Prov., VI.10.1917, 1 male NEW COUNTRY RECORD
18°49'16"N, 98°55'11"E	*Peltonotus morio*	Jameson and Wada 2004	Thailand, Doi Suthep
27°18'42"N, 88°35'57"E	*Peltonotus morio*	Jameson and Wada 2004	Sikkim, India
24°39'32"N, 93°54'22"E	*Peltonotus morio*	Jameson and Wada 2004	India, Manipur
25°22'05"N, 91°45'13"E	*Peltonotus morio*	Jameson and Wada 2004	India, Meghalaya, Khasi Hills
27°09'33"N, 88°36'56"E	*Peltonotus morio*	Jameson and Wada 2004	India, Pedong
27°02'09"N, 88°14'08"E	*Peltonotus morio*	Jameson and Wada 2004	India, Darjeeling
28°16'N, 84°05'E	*Peltonotus morio*	Jameson and Wada 2004	Nepal, Chhachok
14°48'00"N, 106°49'59"E	*Peltonotus nasutus*	FUJI	S. Laos, Attapu, V.13.2007, 1 male, 1 female, ele 450m
14°88'N, 105°87'E	*Peltonotus nasutus*	FUJI	S. Laos, Champasak Province, 2 females,
16°42'18"N, 98°20'44"E	*Peltonotus nasutus*	FUJI	S. Burma, Mt. Dawna, V.1992, 1 female
18°38'31"N, 94°42'56"E	*Peltonotus nasutus*	FUJI	Myanmar, Arakan Province, Nianjyo, 1070m, 1 male, 1 female
15°N, 98°32'E	*Peltonotus nasutus*	BMNH	W. Thailand, Kanchanaburi Prov., Thung Yai Wildlife Sanctuary, mixed riverside forest, M. Brendell, V.8.1988, 10 specimens, within spathe of *Amorphophallus* inflorescence
19°25'N, 103°30'E	*Peltonotus nasutus*	BMNH	Laos, Xiankhouang Prov. V.18.1919, 1 male
26°51'22"N, 104°13'59"E	*Peltonotus nasutus*	Drumont	Chine, Guizhou, Mt. Ping-Qing-Liang-Zi, Weining county, 1-10/VII-2009, 1 male, 3 female NEW COUNTRY RECORD
23°28'5"N, 100°41'E	*Peltonotus nasutus*	Drumont	Chine, Yunnan, Mt. Longtanshan, Jinggu county, VI.11-20, 3 male, Col. Li Jingke NEW COUNTRY RECORD
22°35'N, 99°33'E	*Peltonotus nasutus*	Drumont	Chine, Yunnan, Mt. Daheishan, Menglian county, V.20-31-2009, Col. Li Jingke, 1 female NEW COUNTRY RECORD
22°47'56"N, 108°19'44"E	*Peltonotus nasutus*	AREC	China, Guangxi Zhuang Automonus Region NEW COUNTRY RECORD
17°28'59"N, 101°4'0"E	*Peltonotus nasutus*	IRSBN	Thailand, Changwat Loei, Na Haeo Bio. Sta., V-15-19-2003, light trap, Col. Constant, Smets, and Grootaert, 1 male, 2 female
17°28'59"N, 101°4'0"E	*Peltonotus nasutus*	IRSBN	Thailand, Changwat Loei, Na Haeo Bio. Sta., V.17.2003, edge pond, Col. Constant and Smets, 2 female
17°28'59"N, 101°4'0"E	*Peltonotus nasutus*	IRSBN	Thailand, Changwat Loei, Na Haeo Bio. Sta., V.5-12-2001, light trap, Col. Constant and Grootaert, 2 female
19°27'N, 98°20'E	*Peltonotus nasutus*	IRSBN	N. Thailand, Mae Hong Son Prov., 600 m, 28-V to 2-VI-1999, Col. D. Hauck, 2 male, 2 female
14°16'07"N, 98°59'12"E	*Peltonotus nasutus*	IRSBN	Thailand, Kanchanaburi Prov., Sai Yok NP, VI.4–5.2003, Constant and Smets, 1 male, 1 female
13°49'59"N, 106°57'0"E	*Peltonotus nasutus*	IRSBN	Cambodia, Ratanakiri Prov., Phumi Kalai Thum., VI.1-19.2007, Col. Li Jingke, 1 male, 2 female NEW COUNTRY RECORD
12°18'09"N, 102°59'20"E	*Peltonotus nasutus*	IRSBN	Cambodia, Pursat Prov., Phnum Samkos Wildlife Sanctuary, XI.15, 2005, light trapping, col. Smets and Van, 5 male, 4 female NEW COUNTRY RECORD
12°18'09"N, 102°59'20"E	*Peltonotus nasutus*	IRSBN	Cambodia, Pursat Prov., Phnum Samkos Wildlife Sanctuary, IV.13-14, 2005, light trapping, primary forest edge, col. Smets and Van, 1 female, 1 male,
12°18'09"N, 102°59'20"E	*Peltonotus nasutus*	IRSBN	Cambodia, Pursat Prov., Phnum Samkos Wildlife Sanctuary, IV.16, 2005, light trapping, col. Smets and Van, 3 female, 1 male NEW COUNTRY RECORD
12°18'09"N, 102°59'20"E	*Peltonotus nasutus*	IRSBN	Cambodia, Pursat Prov., Phnum Samkos Wildlife Sanctuary, IV.15, 2005, light trapping, col. Smets and Van, 1 female NEW COUNTRY RECORD
12°51'2"N, 102°36'34"E	*Peltonotus nasutus*	Drumont	Cambodia, Pailin Prov., 270m, V.6-16.2008, col. Murzin, 2 female NEW COUNTRY RECORD
21°17'13"N, 101°10'02"E	*Peltonotus nasutus*	IRSBN	NW Laos, Louang Namtha Prov., Muang Sing, Houaylong-Kao, VI.2-19.2010, 6 male, 16 female
17°58'0"N, 102°35'59"E	*Peltonotus nasutus*	IRSBN	Laos, Vientiane Prov., IV.4-1915, 1 female
17°58'0"N, 102°35'59"E	*Peltonotus nasutus*	IRSBN	Laos, Vientiane Prov., V.18-1915, 1 male
20°09'0"N, 101°19'53"E	*Peltonotus nasutus*	Li et al. 2012	Laos, Bokeo Prov., Pha Ngam
16°46'30"N, 102°37'10"E	*Peltonotus nasutus*	Jameson and Wada 2004	Thailand, Khorat
14°35'21"N, 98°44'29"E	*Peltonotus nasutus*	Jameson and Wada 2004	Thailand, Pu Nam Long Hot Spring
14°47'53"N, 98°44'29"E	*Peltonotus nasutus*	Jameson and Wada 2004	Thailand, Khao Leam Dam
19°05'47"N, 100°57'09"E	*Peltonotus nasutus*	Jameson and Wada 2004	Thailand, Nan Province
19°21'46"N, 98°59'01"E	*Peltonotus nasutus*	Jameson and Wada 2004	Thailand, Ban Chiang Dao
14°43'02"N, 102°01'23"E	*Peltonotus nasutus*	Jameson and Wada 2004	Thailand, Khorat Prov., Pak Thong Chai
17°57'46"N, 102°36'54"E	*Peltonotus nasutus*	Jameson and Wada 2004	Laos, Vientane
19°36'41"N, 103°43'44"E	*Peltonotus nasutus*	Jameson and Wada 2004	Laos, Xiangkhouang
22°20'59"N, 96°55'00"E	*Peltonotus nasutus*	Jameson and Wada 2004	Myanmar, Gokhteik
21°14'14"N, 106°22'34"E	*Peltonotus nasutus*	Jameson and Wada 2004	Vietnam, Tonkin (north Vietnam)
10°44'57"N, 106°40'43"E	*Peltonotus nasutus*	Jameson and Wada 2004	Vietnam, Cochinchina (southern Vietnam)

During the course of our research, we discovered two unrecorded paralectotype specimens. The male lectotype (at BMNH) and eight paralectotypes (6 at BMNH, 2 at MNHN) were previously designated (Jameson and Wada 2004). Two additional paralectotypes (1 male, 1 female) were found at IRSNB. The paralectotype male at IRSNB is labeled: a) “Cochinchina” (handwritten), b) “Collection E. Candèze” (type set with scribed, black box), c) “Type” (type set, red ink, with scribed, black box), d) “Peltonotus nasutus, Arrow co-type” (handwritten), e) “Peltonotus nasutus Type Arrow det Arrow 1908” (handwritten and type set), f) our paralectotype label. The paralectotype female at IRSNB is labeled: a) “Cochinch” (handwritten), b) “Collection E. Candèze” (type set with scribed, black box), c) “Type” (type set, red ink, with scribed, black box), d) “Peltonotus nasutus Type Arrow det Arrow 1908” (handwritten and type set), e) our paralectotype label.

### New distributional records and description of first female specimen for *Peltonotus favonius*

*Peltonotus favonius* Jameson and Wada (Fig. 4) was previously known based only on one male specimen from Vietnam (Jameson and Wada 2009). This species is most similar to *Peltonotus pruinosus*, a species for which only the female holotype is known. The discovery of additional male specimens and the first female specimens facilitates identification of the species, expands the characteristics of the species, and broadens our understanding of the distribution of the species. **New Country Record**(2 male and 2 female specimens deposited in MLJC): **MYANMAR,** Mt. Nweezin, ~750m, 10 km NNE of Puta-o, North Kachin, June 16–21, 1998. The new record extends the known range of the species over 2000 km from Vietnam to Myanmar. Specimens were provided by Shinji Nagai. Male specimens from Myanmar (n=2) possess black and reddish-brown elytra (the holotype specimen from Vietnam possessed black elytra). Female specimens (n=2) differ from the male specimens in the following respects: Color: Head, pronotum, scutellum, propygidium, pygidium, and venter shining black; elytra black or dark reddish-brown with iridescent bloom. Elytron: Epipleuron in ventral view (Fig. 75) broadly expanded from base to apex of metacoxa, weakly convex, not incised at apex, with sparse, setose punctures, setae reddish, moderately long; in dorsal view expansion not developed (lacking dorsal pillow), instead with concave groove adjacent to epipleuron. Propygidium: Surface moderately densely punctate; punctures simple and ocellate, mixed, not setigerous. Pygidium: Surface moderately densely punctate; punctures simple and ocellate, not setigerous. Legs: Protibia tridentate. Proclaws of female 3/4 length of protarsomere 5, claw angled ventrally.

### New distributional records for *Peltonotus morio*

*Peltonotus morio* Burmeister (Fig. 12) is the type species for the genus *Peltonotus* and is one of the most wide-spread species in the genus (Fig. 92). It is distinguished from its close congener, *Peltonotus nasutus* Arrow (Figs 14–15), by its incomplete pronotal basal bead (complete in *Peltonotus nasutus*), form of the male parameres (Figs 65–66), lack of a small tubercle at the apex of the clypeus in the male (Fig. 24) (present in *Peltonotus nasutus* [Fig. 23]), and form of the epipleuron in females (Figs 80 versus 82).

The species is found in northeastern India, Nepal, Bhutan, and Thailand (Jameson and Wada 2004). It can be collected at lights (Dhoj et al. 2009). Within the Palearctic region (Löbl and Smetana 2003) or Sino-Japanese region (Holt et al. 2013), it is the only recorded species of Cyclocephalini (Dynastinae), and it was recorded from Bhutan, Nepal, and Sikkim (Krell 2006). Examination of additional specimens provided **new country records** for *Peltonotus morio* in Myanmar and Vietnam. **New Country Record: MYANMAR** (2 specimens deposited in FUJI): Tanintharyi (near Tenasserim), May-1992, 1 male; Mt. Dawna, May-1992, 763 m elevation, 1 female. **New Country Record: VIETNAM** (1 specimen deposited in IRSNB): Lào Cai Province, June 10, 1917, 1 male. Despite the antiquity of the specimen (nearly 100 years old), the new record in Vietnam extends the known range of the species over 600 km from northern Thailand to northern Vietnam. Based on these distributional data, *Peltonotus morio* and *Peltonotus nasutus* may be narrowly sympatric in southern Myanmar and Thailand.

### Key to Male *Peltonotus* Species

Males: Protibial claws with one claw enlarged and expanded; elytral epipleuron not developed in ventral view. Males of *Peltonotus kyojinus*, *Peltonotus nethis*, *Peltonotus pruinosus*, *Peltonotus suehirogarus*, *Peltonotus mushiyaus*, and *Peltonotus tigerus* are not known.

**Table:** 

1	Apical half of mentum acute, triangular (e.g., Figs 25, 34–35)	2
–	Apical half of mentum rounded (Figs 26–29, 31–33) or quadrate (Fig. 30)	4
2	Punctures of frons and clypeus unisetigerous; parameres as in Fig. 71	*Peltonotus talangensis* Jameson & Jakl
–	Punctures of frons and clypeus multisetigerous (at least laterally); parameres not as in Fig. 71	3
3	Smaller protarsal claw deeply arcuate (Fig. 54); parameres as in Fig. 59	*Peltonotus deltamentum* Jameson & Wada
–	Smaller protarsal claw simply arched; parameres as in Fig. 70	*Peltonotus sisyrus* Jameson & Wada
4	Apex of labrum weakly sinuate (Figs 23–24)	5
–	Apex of labrum bi-emarginate (Figs 21–22) to deeply bilobed (Fig. 20)	6
5	Protibia tridentate with well-developed basal tooth (e.g., Fig. 46); apex of clypeus at middle with tubercle (Fig. 23); parameres as in Fig. 66	*Peltonotus nasutus* Arrow
–	Protibia tridentate with weakly developed basal tooth (e.g., Fig. 49); apex of clypeus lacking tubercle (Fig. 24); parameres as in Fig. 65	*Peltonotus morio* Burmeister
6	Labrum with apex deeply bilobed (e.g.,Fig. 20)	7
–	Labrum with apex bi-emarginate (Figs 21–22)	10
7	Mala of maxilla with setae thick and strongly flattened (with well developed lamellate setal brush); Borneo, Malaysia, and Sumatra; parameres not as in Fig. 63	8
–	Mala of maxilla with setae not thick and strongly flattened (lacking well developed lamellate setal brush) (Fig. 40); South Vietnam; parameres as in Fig. 63	*Peltonotus karubei* Muramoto
8	Punctures of frons lacking setae; parameres as in Fig. 57	*Peltonotus brunnipennis* Benderitter
–	Punctures of frons with dense, velutinous and/or moderately long setae; parameres not as in Fig. 57	9
9	Protarsus with larger claw gracile, subequal at middle and base; maxillary stipes with setae curly at apex (e.g.,Fig. 41); Sarawak	*Peltonotus gracilipodus* Jameson & Wada
–	Protarsus with larger claw robust, much wider at middle than at base; maxillary stipes with setae straight, not curly at apex; Malaysia (Cameron Highlands)	*Peltonotus podocrassus* Jameson & Wada
10	Labial palpomere 2 greatly enlarged and dorsoventrally flattened, 2–3 times wider than apical palpomere 1 (Fig. 28)	11
–	Labial palpomere 2 not greatly enlarged and flattened, less than 1.5 times wider than apical palpomere 1 (Fig. 33)	13
11	Maxillary stipes with setae curly at apex (e.g., Fig. 36); parameres not as in Fig. 68	12
–	Maxillary stipes with setae straight, not curly at apex; parameres as in Fig. 68	*Peltonotus silvanus* Jameson & Wada
12	Elytral color reddish, lighter in color than pronotum and scutellum; punctures of pygidium multisetigerous, setae minute and moderate in length; parameres as in Fig. 64	*Peltonotus malayensis* Arrow
–	Elytral color castaneous, similar in color to pronotum and scutellum (Fig. 1); punctures of pygidium unisetigerous, setae moderate in length; parameres as in Fig. 56	*Peltonotus animus* Jameson & Wada
13	Protibia tridentate, basal tooth well developed or weakly developed (Figs 46, 48, 49)	14
–	Protibia bidentate (Fig. 47, 51)	15
14	Protibia with basal tooth well developed (Figs 46, 48), external margin without velutinous setae from middle to near base; parameres as in Fig. 61	*Peltonotus fujiokai* Jameson & Wada
–	Protibia externally with basal tooth weakly developed (Fig. 49), external margin with velutinous setae from middle to near base; parameres as in Fig. 67	*Peltonotus rubripennis* Miyake & Yamaya
15	Elytra reddish with castaneous vittae (Figs 18–19); parameres as in Fig. 72	*Peltonotus vittatus* Arrow
–	Elytra lacking vittae, entirely reddish, castaneous, or black; parameres not as in Fig. 72	16
16	Pronotal basal bead lacking, terminating at basolateral angle; length less than 15.0 mm; parameres as in Fig. 60	*Peltonotus favonius* Jameson & Wada
–	Pronotal basal bead present, extending beyond basolateral angle (obscured anterior to scutellum); length greater than 17.0 mm; parameres not as in Fig. 60	17
17	Protarsomere 5 with well-developed internoapical protrusion (Fig. 50), lacking weak medial protrusion; region surrounding Mt. Bawang, Kalimantan	*Peltonotus adelphosimilis* Jameson & Wada
–	Protarsomere 5 lacking internoapical protrusion; weak protrusion at middle (Fig. 53); Sabah	*Peltonotus similis* Arrow

### Key to Female *Peltonotus* Species

Females: Protibial claws similar in size and shape; elytral epipleuron developed or simple in ventral view. Females of *Peltonotus deltamentum*, *Peltonotus karubei*, and *Peltonotus animus* are not known.

**Table:** 

1	Apical half of mentum acute, triangular (Figs 25, 34–35)	2
–	Apical half of mentum rounded (Figs 26–29, 31–33) or quadrate (Fig. 30)	3
2	Punctures of frons and clypeus multisetigerous	*Peltonotus sisyrus* Jameson & Wada
–	Punctures of frons and clypeus unisetigerous	*Peltonotus talangensis* Jameson & Jakl
3	Apex of labrum weakly sinuate (Figs 23–24)	4
–	Apex of labrum bi-emarginate (Figs 21–22) to deeply bilobed (Fig. 20)	5
4	Apex of clypeus with weak, medial tubercle; lateral pillow of elytron (dorsal view) elongate-oval, extending more than half length of epipleuron; epipleuron as in Fig. 82	*Peltonotus nasutus* Arrow
–	Apex of clypeus lacking weak tubercle; lateral pillow of elytron (dorsal view) narrower at apex and broader at base, extending less than half length of epipleuron; epipleuron as in Fig. 80	*Peltonotus morio* Burmeister
5	Elytra with castaneous vittae or maculae (e.g., Figs 13, 18–19)	6
–	Elytra lacking vittae, entirely castaneous, reddish, or black	7
6	Elytral epipleuron in ventral view simple, lacking apical incision (Fig. 81)	*Peltonotus mushiyaus* Jameson & Wada
–	Elytral epipleuron in ventral view incised at apex (Fig. 91)	*Peltonotus vittatus* Arrow
7	Labrum with apex deeply bilobed (e.g., Fig. 20)	8
–	Labrum with apex bi-emarginate (e.g.,Figs 21–22)	13
8	Elytral epipleuron in ventral view simple, not emarginated (Fig. 83)	*Peltonotus nethis* Jameson & Wada
–	Elytral epipleuron in ventral view emarginated (e.g., Fig. 73)	9
9	Maxillary stipes with setae curly at apex (e.g., Fig. 41)	10
–	Maxillary stipes with setae straight, not curly at apex	11
10	Epipleural emargination with well-developed tooth in ventral view (Fig. 73)	*Peltonotus brunnipennis* Benderitter
–	Epipleural emargination with moderately developed tooth in ventral view (Fig. 77)	*Peltonotus gracilipodus* Jameson & Wada
11	Elytra entirely reddish (Fig. 17)	*Peltonotus tigerus* Jameson & Wada
–	Elytra entirely black	12
12	Lateral pillow of elytron (dorsal view) well-developed, extending medially at least ¼ elytral width, visible in ventral view (Fig. 88)	*Peltonotus suehirogarus* Jameson & Wada
–	Lateral pillow of elytron (dorsal view) moderately developed, extending medially about 1/8 elytral width, not visible in ventral view (Fig. 77)	*Peltonotus podocrassus* Jameson & Wada
13	Elytral epipleuron in ventral view broad, nearly parallel from base to near metacoxa, lacking emargination (Fig. 75, 84)	14
–	Elytral epipleuron in ventral view narrowing from base to near metacoxa (not parallel-sided), with or without emargination (e.g., Figs 76, 78–79)	15
14	Elytral epipleuron in ventral view with sparse, reddish, moderately long setae	*Peltonotus favonius* Jameson & Wada
–	Elytral epipleuron in ventral view without setae	*Peltonotus pruinosus* Arrow
15	Labial palpomere 2 greatly enlarged and dorsoventrally flattened, 2–3 times wider than palpomere 1 (e.g., Fig. 28)	16
–	Labial palpomere 2 not greatly enlarged and flattened, at most 1.5 times wider than palpomere 1 (e.g., Fig. 33)	17
16	Maxillary stipes with setae curly at apex (Fig. 41); lateral pillow of elytron (dorsal view) well-developed, visible in ventral view (Fig. 79)	*Peltonotus malayensis* Arrow
–	Maxillary stipes with setae straight, not curly at apex; lateral pillow of elytron (dorsal view) moderately developed, not visible in ventral view (Fig. 77)	*Peltonotus silvanus* Jameson & Wada
17	Body length more than 20 mm; epipleuron in ventral view simple, not emarginate (Fig. 78)	*Peltonotus kyojinus* Jameson & Wada
–	Body length less than 20 mm; epipleuron in ventral view simple or emarginate (Figs 74, 76, 85–86)	18
18	Elytral epipleuron emarginate in ventral view (Fig. 86)	19
–	Elytral epipleuron simple in ventral view (Figs 76, 85)	21
19	Elytral epipleuron in ventral view with round emargination (Figs 74, 86); not occurring in Mt. Bawang, Kalimantan region of Borneo	20
–	Elytral epipleuron in ventral view with elongate-oval emargination; Mt. Bawang, Kalimantan region of Borneo	*Peltonotus adelphosimilis* Jameson & Wada
20	Punctures of frons and clypeus mulitsetigerous, setae minute and/or short; elytral epipleuron as in Fig. 86; Borneo	*Peltonotus similis* Arrow
–	Punctures of frons and clypeus unisetigerous, setae minute; elytral epipleuron as in Fig. 74; Sumatra	*Peltonotus cybele* Jameson & Wada
21	Elytral epipleuron in ventral view terminating near metacoxa (Fig. 85)	*Peltonotus rubripennis* Miyake & Yamaya
–	Elytral epipleuron in ventral view extending posterior of metacoxa, terminating near sternite 3 (Fig. 76) *Peltonotus fujiokai* Jameson & Wada

## *Peltonotus* species diagnoses

### Peltonotus adelphosimilis

Jameson & Wada, 2004

http://species-id.net/wiki/Peltonotus_adelphosimilis

Figs 50 , 55

#### Diagnosis (male and female).

Length 20.3–18.9 mm, color overall black or castaneous, elytra black or castaneous with or without iridescent bloom, head with some multisetigerous punctures, labrum bi-emarginate, mentum rounded in apical half, labial palpomere 2 not enlarged or obviously dorsoventrally flattened, mala lacking lamellate setal brush, maxillary stipes without setae curled at apices, male protibia bidentate, protarsomere 5 of male with internoapical protuberance (Fig. 50), form of parameres (Fig. 55), female epipleuron incised and with rounded emargination (similar to *Peltonotus similis*, Fig. 86).

#### Distribution.

Indonesia, Borneo Island (Kalimantan).

### Peltonotus animus

Jameson & Wada, 2009

http://species-id.net/wiki/Peltonotus_animus

Figs 1 , 36 , 45 , 56

#### Diagnosis (male only).

Length ~16.5 mm, color overall castaneous, elytra castaneous with weak iridescent bloom (Fig. 1), frons with some multisetigerous punctures, labrum bi-emarginate, mentum rounded in apical half, labial palpomere 2 enlarged and dorsoventrally flattened, mala with dense lamellate setal brush (Fig. 36), maxillary stipes with some setae curled at apices (Fig. 36), male protibia tridentate with basal tooth obsolete (Fig. 45), and male parameres (Fig. 56).

#### Distribution.

Indonesia, Sumatra Island.

### Peltonotus brunnipennis

Benderitter, 1934

http://species-id.net/wiki/Peltonotus_brunnipennis

Figs 57 , 73

#### Diagnosis (male and female).

Length 14.5–16.9 mm, color overall castaneous, elytra reddish-orange or black with iridescent bloom, head punctate and lacking setae, labrum deeply bi-lobed, mentum rounded in apical half, labial palpomere 2 enlarged and obviously dorsoventrally flattened, mala with lamellate setal brush, maxillary stipes with some setae curled at apices, male protibia tridentate, form of parameres (Fig. 57), female epipleuron incised and with oval emargination (Fig. 73).

#### Distribution.

Malaysia, Borneo Island (Sabah and Sarawak).

### Peltonotus cybele

Jameson & Wada, 2009

http://species-id.net/wiki/Peltonotus_cybele

Figs 2–3 , 37 , 46 , 58 , 74

#### Diagnosis (male and female).

Length 14.5–16.5 mm, color overall castaneous, elytra castaneous suffused with dark red or reddish-brown and iridescent bloom (Figs 2–3), head with some unisetigerous punctures, labrum bi-emarginate, mentum rounded in apical half, labial palpomere 2 not enlarged or obviously dorsoventrally flattened, mala lacking lamellate setal brush (Fig. 37), maxillary stipes without setae curled at apices (Fig. 37), male protibia tridentate (Fig. 46), form of parameres (Fig. 58), female epipleuron incised and with rounded emargination (Fig. 74).

#### Distribution.

Indonesia, Sumatra Island.

### Peltonotus deltamentum

Jameson & Wada, 2004

http://species-id.net/wiki/Peltonotus_deltamentum

Figs 25 , 38 , 54 , 59

#### Diagnosis (male only).

Length ~16.6 mm, color overall castaneous, elytra castaneous with weak iridescent bloom, head with some multisetigerous punctures, labrum bi-emarginate, mentum triangular in apical half (Fig. 25), labial palpomere 2 enlarged and dorsoventrally flattened, mala with dense lamellate setal brush (Fig. 38), maxillary stipes with setae curled at apices (Fig. 38), male protibia tridentate with basal tooth weakly developed, male proclaw strongly arcuate in ventral view (Fig. 54), form of parameres (Fig. 59).

#### Distribution.

Indonesia, Borneo Island (Kalimantan).

### Peltonotus favonius

Jameson & Wada, 2009

http://species-id.net/wiki/Peltonotus_favonius

Figs 4 , 39 , 51 , 60 , 75

#### Diagnosis (male and female).

Length ~14.6 mm, color overall black, elytra black or dark reddish brown with iridescent bloom (Fig. 4), head with simple punctures (lacking setae), labrum bi-emarginate, mentum rounded in apical half, labial palpomere 2 not enlarged or obviously dorsoventrally flattened, mala lacking lamellate setal brush (Fig. 39), maxillary stipes without setae curled at apices (Fig. 39), male protibia bidentate (Fig. 51), form of parameres (Fig. 60), female epipleuron broadly expanded, weakly convex, extending from base to metacoxa, lacking incised apex (Fig. 75).

#### Distribution.

Vietnam and Myanmar.

#### Remarks.

This species is most similar to *Peltonotus pruinosus*, a species for which only the female holotype is known. Previously, this species was only known from the male holotype specimen from Vietnam.

### Peltonotus fujiokai

Jameson & Wada, 2004

http://species-id.net/wiki/Peltonotus_fujiokai

Figs 5–8 , 61 , 76

#### Diagnosis (male and female).

Length 14.1–14.6 mm, color overall castaneous, elytra reddish-brown with castaneous vittae, reddish-brown, or black with iridescent bloom (Figs 5–8), head with some unisetigerous punctures, labrum bi-emarginate, mentum rounded in apical half, labial palpomere 2 not enlarged and not dorsoventrally flattened, mala without dense lamellate setal brush, maxillary stipes without setae curled at apices, male protibia tridentate, form of parameres (Fig. 61), female epipleuron simple, not incised and lacking emargination (Fig. 76).

#### Distribution.

Indonesia, Borneo Island (Kalimantan); Malaysia, Borneo Island (Sabah).

### Peltonotus gracilipodus

Jameson & Wada, 2004

http://species-id.net/wiki/Peltonotus_gracilipodus

Figs 26 , 62 , 77

#### Diagnosis (male and female).

Length 14.4–16.8 mm, color overall castaneous, elytra castaneous with weak iridescent bloom, head with some multisetigerous punctures, labrum deeply bi-lobed, mentum rounded in apical half (Fig. 26), labial palpomere 2 enlarged and obviously dorsoventrally flattened (Fig. 26), mala with lamellate setal brush, maxillary stipes with some setae curled at apices, male protibia bidentate, form of parameres (Fig. 62), female epipleuron incised and with oblong-oval emargination (Fig. 77).

#### Distribution.

Indonesia, Sumatra Island.

#### Remarks.

*Peltonotus gracilipodus* and *Peltonotus podocrassus* (distributed in peninsular Malaysia) have quite similar male parameres and females have quite similar epipleura, perhaps indicating recent isolation of ancestral populations.

### Peltonotus karubei

Muramoto, 2000

http://species-id.net/wiki/Peltonotus_karubei

Figs 9 , 20 , 40 , 63

#### Diagnosis (male only).

Length 13.4–14.5 mm, overall color black or castaneous, elytra reddish orange or black with iridescent bloom (Fig. 9), head with some multisetigerous punctures, labrum deeply bilobed (Fig. 20), labial palpomere 2 enlarged and obviously dorsoventrally flattened (Fig. 40), mala with weak lamellate setal brush (Fig. 40), maxillary stipes without setae curled at apices, male protibia bidentate, form of male parameres (Fig. 63).

#### Distribution.

Vietnam (southern).

### Peltonotus kyojinus

Jameson & Wada, 2004

http://species-id.net/wiki/Peltonotus_kyojinus

Figs 27 , 78

#### Diagnosis (female only).

Length 21.3 mm, color overall castaneous, elytral disc brown with iridescent bloom, head with some multisetigerous punctures, labrum bi-emarginate, mentum rounded in apical half (Fig. 27), labial palpomere 2 not enlarged and not obviously dorsoventrally flattened, mala without lamellate setal brush, maxillary stipes without setae curled at apices, female epipleuron simple, not incised and lacking emargination (Fig. 78).

#### Distribution.

Indonesia, Borneo Island (Kalimantan).

#### Remarks.

*Peltonotus kyojinus* is the largest species of *Peltonotus*.

### Peltonotus malayensis

Arrow, 1910

http://species-id.net/wiki/Peltonotus_malayensis

Figs 10–11 , 21 , 28 , 41 , 47 , 64 , 79

#### Diagnosis (male and female).

Length 14.4–17.2 mm, color overall castaneous or black, elytra reddish-brown or black with weak iridescent bloom (Figs 10–11), head with some multisetigerous punctures, labrum bi-emarginate (Fig. 21), mentum rounded in apical half (Fig. 28), labial palpomere 2 enlarged and obviously dorsoventrally flattened (Fig. 41), mala with weak lamellate setal brush, maxillary stipes setae curled at apices (Fig. 41), male protibia bidentate (Fig. 47), form of male parameres (Fig. 64), female epipleuron incised and with rounded emargination (Fig. 79).

#### Distribution.

Brunei; Indonesia, Borneo Island (Kalimantan); Malaysia, Borneo Island (Sarawak).

### Peltonotus morio

Burmeister, 1847

http://species-id.net/wiki/Peltonotus_morio

Figs 12 , 24 , 65 , 80 , 92

#### Diagnosis (male and female).

Length 14.0–18.0 mm, color overall black or castaneous, elytra black or castaneous and shining (Fig. 12), head with unisetigerous punctures, labrum weakly sinuate (Fig. 24), mentum quadrate in apical half, labial palpomere 2 not enlarged and not dorsoventrally flattened, mala lacking lamellate setal brush, maxillary stipes without setae curled at apices, male protibia tridentate with basal tooth weakly developed, form of male parameres (Fig. 65), female epipleuron weakly, quadrately incised (Fig. 80) and with moderately developed dorsal pillow.

#### Distribution

(Fig. 92). Bhutan, India (northeastern), Myanmar, Nepal, Thailand, Vietnam.

### Peltonotus mushiyaus

Jameson & Wada, 2009

http://species-id.net/wiki/Peltonotus_mushiyaus

Figs 13 , 42 , 81

#### Diagnosis (female only).

Length ~11.8 mm, overall color castaneous, elytral disc orangish-tan with castaneous maculae and iridescent bloom (Fig. 13), head with some unisetigerous punctures, labrum bi-emarginate, mentum rounded in apical half, labial palpomere 2 not enlarged or obviously dorsoventrally flattened, mala lacking lamellate setal brush (Fig. 42), maxillary stipes without setae curled at apices (Fig. 42), female epipleuron simple, not expanded (Fig. 81).

#### Distribution.

Malaysia, Borneo Island (Sabah).

#### Remarks.

*Peltonotus mushiyaus* is the smallest species in the genus. We hypothesize that males of this species will possess orangish-tan elytra with castaneous maculae, similar to males of *Peltonotus vittatus*.

### Peltonotus nasutus

Arrow, 1910

http://species-id.net/wiki/Peltonotus_nasutus

Figs 14–15 , 23 , 30 , 52 , 66 , 82 , 92

#### Diagnosis (male and female).

Length 19.6–20.6 mm, color overall black or castaneous, elytra black or castaneous and shining (Fig. 14–15), head with unisetigerous punctures and apex of clypeus with weak tubercle medially (Fig. 23), labrum weakly sinuate (Fig. 23), mentum quadrate in apical half (Fig. 30), labial palpomere 2 not enlarged and not dorsoventrally flattened, mala lacking lamellate setal brush, maxillary stipes without setae curled at apices, male protibia tridentate with well developed basal tooth, male protibial claw greatly enlarged (Fig. 52), form of male parameres (Fig. 66), female epipleuron weakly, quadrately incised (Fig. 82) and with well developed dorsal pillow.

#### Distribution

(Fig. 92). Cambodia, China (southern), Laos, Myanmar, Thailand, Vietnam.

#### Remarks.

*Peltonotus nasutus* is the most common species in the genus and the only species with an apicomedial tubercle on the clypeus (male only).

### Peltonotus nethis

Jameson & Wada, 2004

http://species-id.net/wiki/Peltonotus_nethis

Figs 31 , 83

#### Diagnosis (female only).

Length ~13.7 mm, color overall black, elytra black with iridescent bloom, head with unisetigerous punctures or lacking setae, labrum bi-emarginate, mentum rounded in apical half (Fig. 31), labial palpomere 2 greatly enlarged and dorsoventrally flattened, mala with lamellate setal brush, maxillary stipes without setae curled at apices, female epipleuron simple, not incised (Fig. 83).

#### Distribution.

Malaysia, Borneo Island (Sabah).

### Peltonotus podocrassus

Jameson & Wada, 2004

http://species-id.net/wiki/Peltonotus_podocrassus

Figs 29 , 62 , 77

#### Diagnosis (male and female).

Length 17.6–18.7 mm, color overall castaneous, elytra castaneous with weak iridescent bloom, head with some multisetigerous punctures, labrum deeply bi-lobed, mentum rounded in apical half (Fig. 29), labial palpomere 2 enlarged and obviously dorsoventrally flattened (Fig. 29), mala with lamellate setal brush, maxillary stipes lacking setae curled at apices, male protibia bidentate, form of parameres (Fig. 62), female epipleuron incised and with oblong-oval emargination (Fig. 77).

#### Distribution.

Malaysia (Peninsular Malaysia).

#### Remarks.

*Peltonotus podocrassus* and *Peltonotus gracilipodus* (distributed in Sumatra) are similar with respect to the male parameres and female epipleura. This may be indicative of recent divergence from a common ancestor.

### Peltonotus pruinosus

Arrow, 1910

http://species-id.net/wiki/Peltonotus_pruinosus

Figs 32 , 84

#### Diagnosis (female only).

Length ~15.7 mm, color overall black, elytra black with iridescent bloom, head punctate and lacking setae, labrum bi-emarginate, mentum rounded in apical half and moderately bi-lobed at middle (Fig. 32), labial palpomere 2 not enlarged and not obviously dorsoventrally flattened (Fig. 32), mala without lamellate setal brush, maxillary stipes without setae curled at apices, female epipleuron broadly expanded and lacking emargination at apex (Fig. 84).

#### Distribution.

India.

### Peltonotus rubripennis

Miyake & Yamaya, 1994

http://species-id.net/wiki/Peltonotus_rubripennis

Figs 49 , 67 , 85

#### Diagnosis (male and female).

Length 12.0–12.5 mm, color overall castaneous, elytral disc brown with iridescent bloom, head with unisetigerous punctures, labrum bi-emarginate, mentum rounded in apical half, labial palpomere 2 slightly enlarged and not obviously dorsoventrally flattened, mala lacking lamellate setal brush, maxillary stipes lacking setae curled at apices, male protibia tridentate with basal tooth weakly developed (Fig. 49), form of parameres (Fig. 67), female epipleuron simple and lacking emargination at apex (Fig. 85).

#### Distribution.

Malaysia, Borneo Island (Sabah and Sarawak).

### Peltonotus silvanus

Jameson & Wada, 2004

http://species-id.net/wiki/Peltonotus_silvanus

Figs 68 , 77

#### Diagnosis (male and female).

Length 16.3–17.8 mm, color overall castaneous, elytra castaneous, dark-brown, or black with weak iridescent bloom, head with some multisetigerous punctures, labrum bi-emarginate, mentum rounded in apical half, labial palpomere 2 enlarged and obviously dorsoventrally flattened, mala with lamellate setal brush, maxillary stipes lacking setae curled at apices, male protarsomeres 2–4 with apices expanded, male protibia bidentate, form of parameres (Fig. 68), female epipleuron incised and with oblong-oval emargination (Fig. 77).

#### Distribution.

Indonesia, Borneo Island (Kalimantan); Malaysia, Borneo Island (Sarawak).

### Peltonotus similis

Arrow, 1931

http://species-id.net/wiki/Peltonotus_similis

Figs 33 , 53 , 69 , 86

#### Diagnosis (male and female).

Length 18.0–20.9 mm, color overall dark brown or black, elytra dark brown or black with or without iridescent bloom, head with some multisetigerous punctures, labrum bi-emarginate, mentum rounded in apical half (Fig. 33), labial palpomere 2 slightly enlarged and not obviously dorsoventrally flattened (Fig. 33), mala without lamellate setal brush, maxillary stipes without setae curled at apices, protarsomere 5 of male with internomedial protuberance (Fig. 53), male protibia bidentate, form of parameres (Fig. 69), female epipleuron incised and with rounded emargination (Fig. 86).

#### Distribution.

Malaysia, Borneo Island (Sabah).

### Peltonotus sisyrus

Jameson & Wada, 2004

http://species-id.net/wiki/Peltonotus_sisyrus

Figs 34 , 70 , 87

#### Diagnosis (male and female).

Length 16.1–16.4 mm, overall castaneous, elytra castaneous with weak iridescent bloom, head with some punctures multisetigerous, labrum bi-emarginate, mentum triangular in apical half (Fig. 34), labial palpomere 2 enlarged and obviously dorsoventrally flattened (Fig. 34), mala with lamellate setal brush, maxillary stipes without setae curled at apices, male protibia bidentate, form of parameres (Fig. 70), female epipleuron incised and with broad, elongate emargination (Fig. 87).

#### Distribution.

Indonesia, Sumatra Island.

### Peltonotus suehirogarus

Jameson & Wada, 2004

http://species-id.net/wiki/Peltonotus_suehirogarus

Fig. 88

#### Diagnosis (female only).

Length 16.9–18.0 mm, color overall black, elytra black with iridescent bloom, head with some multisetigerous punctures, labrum bi-emarginate, mentum rounded in apical half, labial palpomere 2 enlarged and obviously dorsoventrally flattened, mala with lamellate setal brush, maxillary stipes with some setae weakly curled at apices, female epipleuron incised and with oblong-oval emargination (Fig. 88).

#### Distribution.

Indonesia, Borneo Island (Kalimantan); Malaysia, Borneo Island (Sarawak).

### Peltonotus talangensis

Jameson & Jakl, 2010

http://species-id.net/wiki/Peltonotus_talangensis

Figs 16 , 35 , 43 , 48 , 71 , 89

#### Diagnosis (male and female).

Length 14.1–15.2 mm, color overall castaneous, elytra castaneous or with weak reddish tones and lacking iridescent bloom (Fig. 16), head with some punctures unisetigerous, labrum bi-emarginate, mentum triangular in apical half (Fig. 35), labial palpomere 2 enlarged and obviously dorsoventrally flattened (Fig. 35), mala with lamellate setal brush (Fig. 43), maxillary stipes without setae curled at apices (Fig. 43), male protibia tridentate (Fig. 48), form of parameres (Fig. 71), female epipleuron simple, not incised (Fig. 89).

#### Distribution.

Indonesia, Sumatra Island.

### Peltonotus tigerus

Jameson & Wada, 2009

http://species-id.net/wiki/Peltonotus_tigerus

Figs 17 , 44 , 90

#### Diagnosis (female only).

Length ~13.7 mm, overall color black or castaneous, elytra reddish-brown with weak iridescent bloom (Fig. 17), head with some punctures multisetigerous, labrum bi-emarginate, labial palpomere 2 enlarged and dorsoventrally flattened, mala with well developed lamellate setal brush (Fig. 44), maxillary stipes without setae curled at apices (Fig. 44), female epipleuron incised with a round or oval emargination (Fig. 90).

#### Distribution.

Thailand.

#### Remarks.

We hypothesize that males of this species will possess reddish-brown elytra, similar to the coloration of the female.

### Peltonotus vittatus

Arrow, 1910

http://species-id.net/wiki/Peltonotus_vittatus

Figs 18–19 , 22 , 72 , 91

#### Diagnosis (male and female).

Length 12.3–14.4 mm, color overall black or castaneous with pronotum reddish or black and with dark discal maculae, elytra reddish and with dark discal maculae and iridescent bloom (Figs 18–19), head with some multisetigerous punctures, labrum bi-emarginate (Fig. 22), mentum rounded in apical half, labial palpomere 2 not enlarged and not obviously dorsoventrally flattened, mala without lamellate setal brush, maxillary stipes without setae curled at apices, male protibia bidentate (or tridentate with basal tooth weakly developed), form of parameres (Fig. 72), female epipleuron narrowly incised (Fig. 91) with well developed dorsal pillow.

#### Distribution.

Malaysia, Borneo Island (Sabah and Sarawak).

## Supplementary Material

XML Treatment for Peltonotus adelphosimilis

XML Treatment for Peltonotus animus

XML Treatment for Peltonotus brunnipennis

XML Treatment for Peltonotus cybele

XML Treatment for Peltonotus deltamentum

XML Treatment for Peltonotus favonius

XML Treatment for Peltonotus fujiokai

XML Treatment for Peltonotus gracilipodus

XML Treatment for Peltonotus karubei

XML Treatment for Peltonotus kyojinus

XML Treatment for Peltonotus malayensis

XML Treatment for Peltonotus morio

XML Treatment for Peltonotus mushiyaus

XML Treatment for Peltonotus nasutus

XML Treatment for Peltonotus nethis

XML Treatment for Peltonotus podocrassus

XML Treatment for Peltonotus pruinosus

XML Treatment for Peltonotus rubripennis

XML Treatment for Peltonotus silvanus

XML Treatment for Peltonotus similis

XML Treatment for Peltonotus sisyrus

XML Treatment for Peltonotus suehirogarus

XML Treatment for Peltonotus talangensis

XML Treatment for Peltonotus tigerus

XML Treatment for Peltonotus vittatus

## Appendix

Supplemental file for dynamic mapping. (doi: 10.3897/zookeys.320.5352.app) Microsoft Excel document (xls).

**Explanation note:** Distribution maps were generated by entering latitude and longitude data into Microsoft Excel 2008 and uploaded to EarthPoint (http://www.earthpoint.us/ExcelToKml.aspx) and GoogleEarth (http://www.google.com/earth/index.html). This supplementary file allows addition of data and interactive mapping or niche modeling. Please note, however, that older localities have a wide margin of error and their lack of precision is not conducive to ecological or niche modeling.
